# Electroanatomic Mapping Clues for Distinguishing a Breakthrough Site from the True Focal Source: Beware of a Big Focus

**DOI:** 10.19102/icrm.2026.17084

**Published:** 2026-08-15

**Authors:** Vatsal Singh, K. Venkadesan, Bhagya Narayan Pandit, Ranjit Kumar Nath

**Affiliations:** 1 Department of Cardiology, Dr. Ram Manohar Lohia Hospital, Atal Bihari Vajpayee Institute of Medical Sciences, New Delhi, India; 2 Department of Cardiology, Himalayan Institute of Medical Science, Swami Rama Himalayan University, Dehradun, India

**Keywords:** Bachmann’s bundle, focal atrial tachycardia, intercaval bundle, right atrial posteromedial connection, right superior pulmonary vein tachycardia

## Abstract

This case highlights an important electroanatomic mapping clue that electrophysiologists should be aware of when ablating focal atrial arrhythmias.

## Case presentation

**Figure 1:**
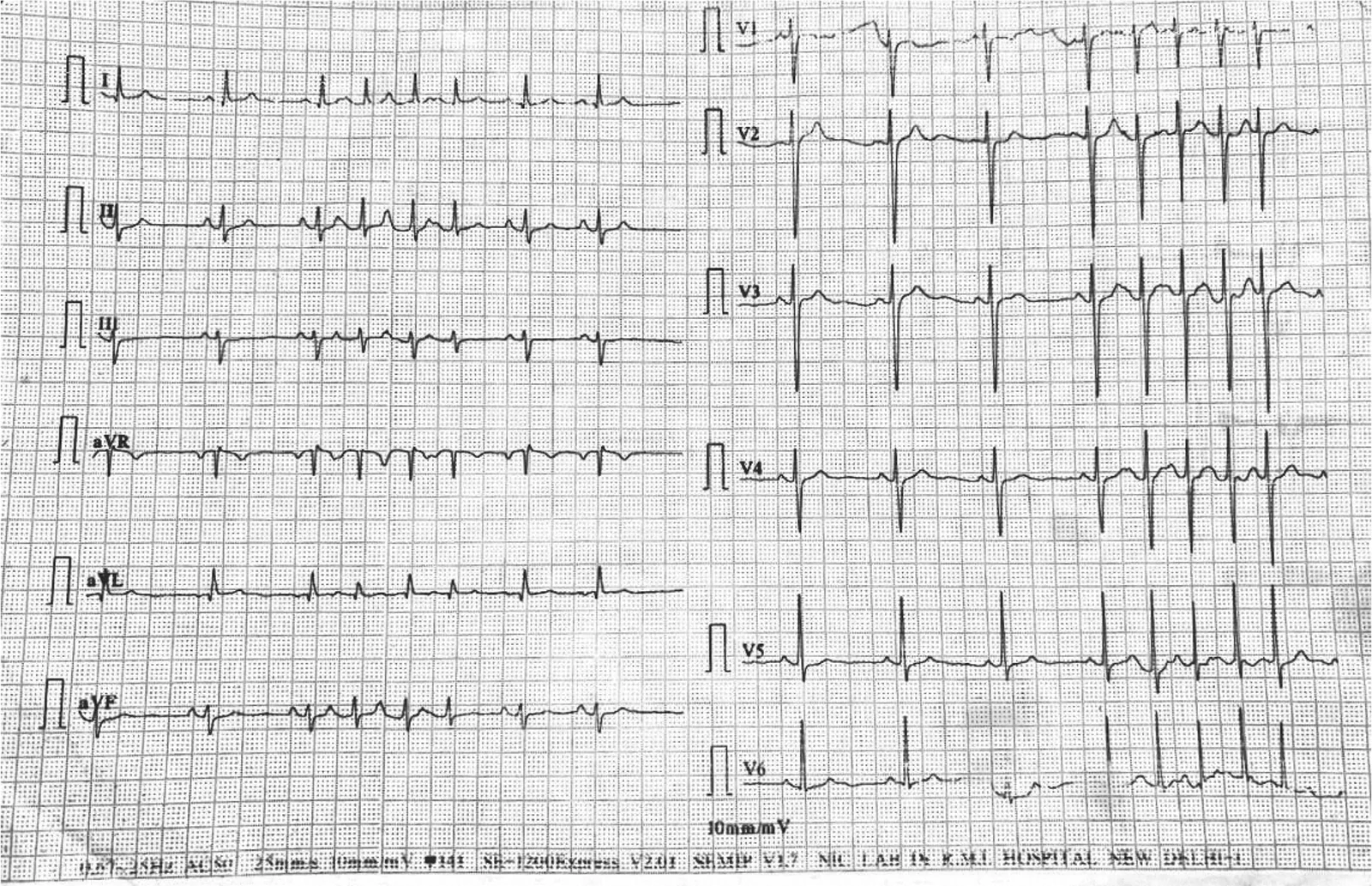
Surface 12-lead electrocardiogram during runs of clinical tachycardia.

**Figure 2:**
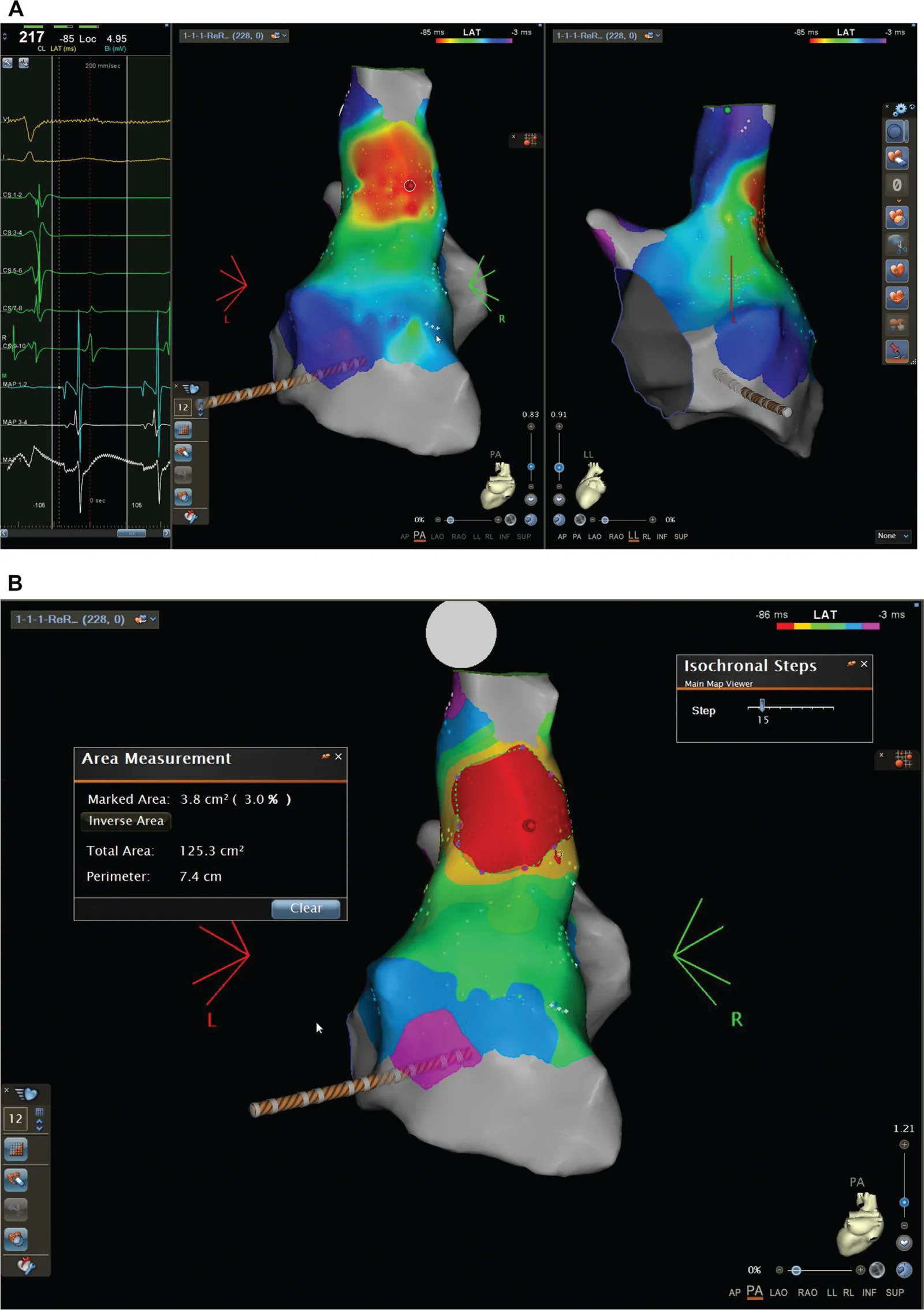
**A:** Right atrial activation map during tachycardia. The red pin marks the site of earliest activation within the right atrium. **B:** Map highlighting the extent of right atrial tissue depolarized within the initial 10 ms of activation (area, ~3.8 cm^2^).

A 22-year-old man presented with a several-year history of incessant palpitations associated with presyncope. A 12-lead electrocardiogram showing tachycardia runs is presented in **Figure 1**. The right atrial (RA) activation map during the tachycardia is shown in **Figures 2A and 2B**. We questioned whether the earliest activation point marked on the electroanatomic map is an appropriate site for ablation.

## Discussion

### Electrophysiological study and mapping

A diagnosis of focal atrial tachycardia was established based on standard electrophysiological criteria. The P-wave morphology was positive in leads V1, I, II, III, and aVF, suggesting a possible focus in the superior crista terminalis region or the right superior pulmonary vein (RSPV) as per the Kistler algorithm.^1^ The initial inspection of the RA activation map during the tachycardia **(Figure 2A)** suggested a focal origin in the posterosuperior RA, with the earliest activation site marked by a red pin. However, the area of early activation was notably broad. Quantitative analysis revealed that the initial 10 ms of RA depolarization encompassed an area of approximately 3.8 cm^2^ **(Figure 2B)**. This wide area of initial activation is atypical for a true focal origin and instead suggests a breakthrough site from an adjacent structure. This finding aligns with prior observations by Long et al., who described an initial depolarization area measuring >3.15 cm^2^ in the RA as a specific marker (sensitivity, 87.5%; specificity, 100%) for an RSPV origin of atrial tachycardia.^2^

**Figure 3:**
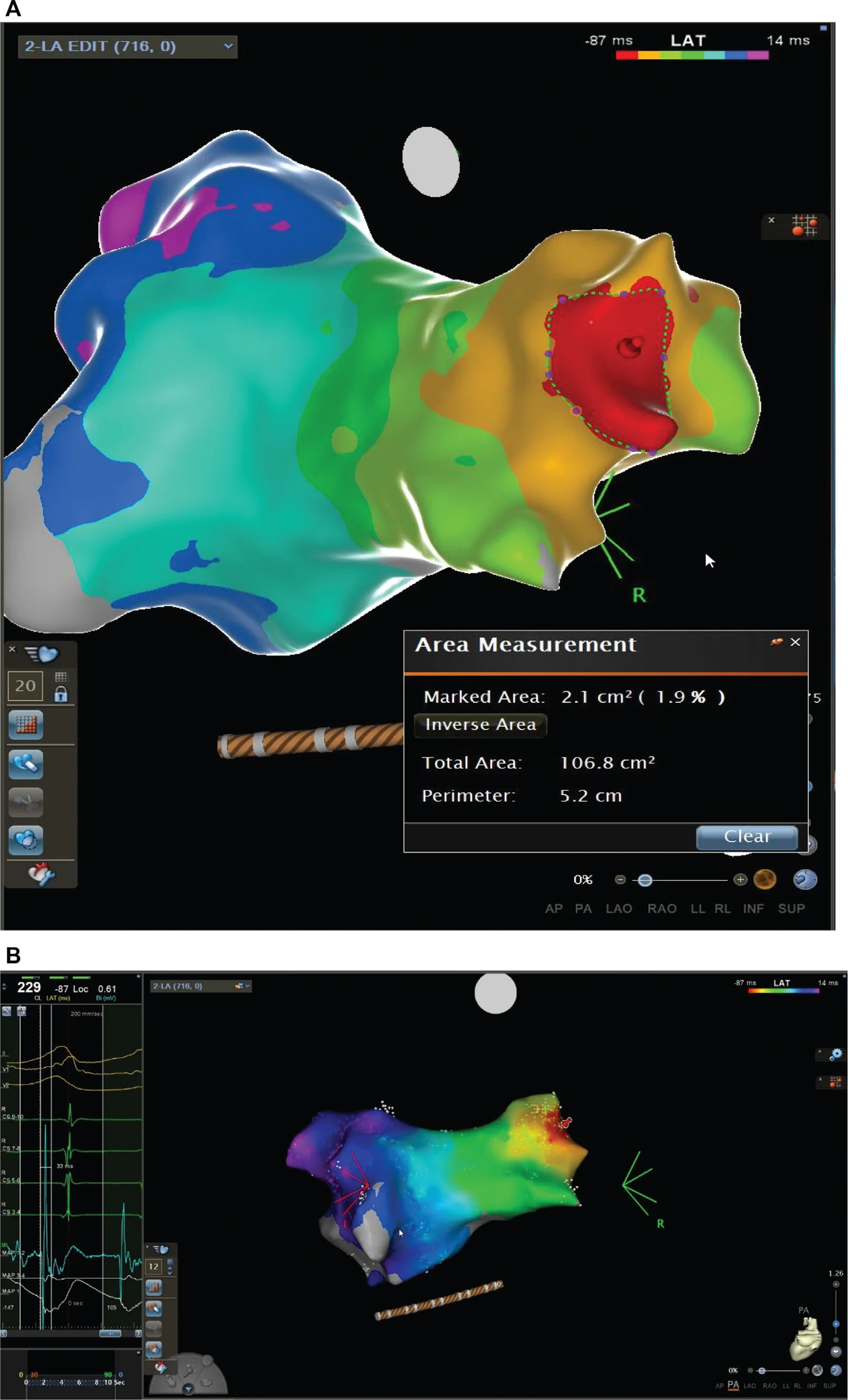
**A:** Left atrial activation map with the earliest site located in the posterior right superior pulmonary vein (RSPV). The area activated in the first 10 ms (~2.1 cm^2^) is shown. **B:** Intracardiac electrograms at the successful ablation site in the RSPV, demonstrating a sharp near-field potential (−33 ms to P-wave onset) with a QS unipolar morphology.

**Figure 4:**
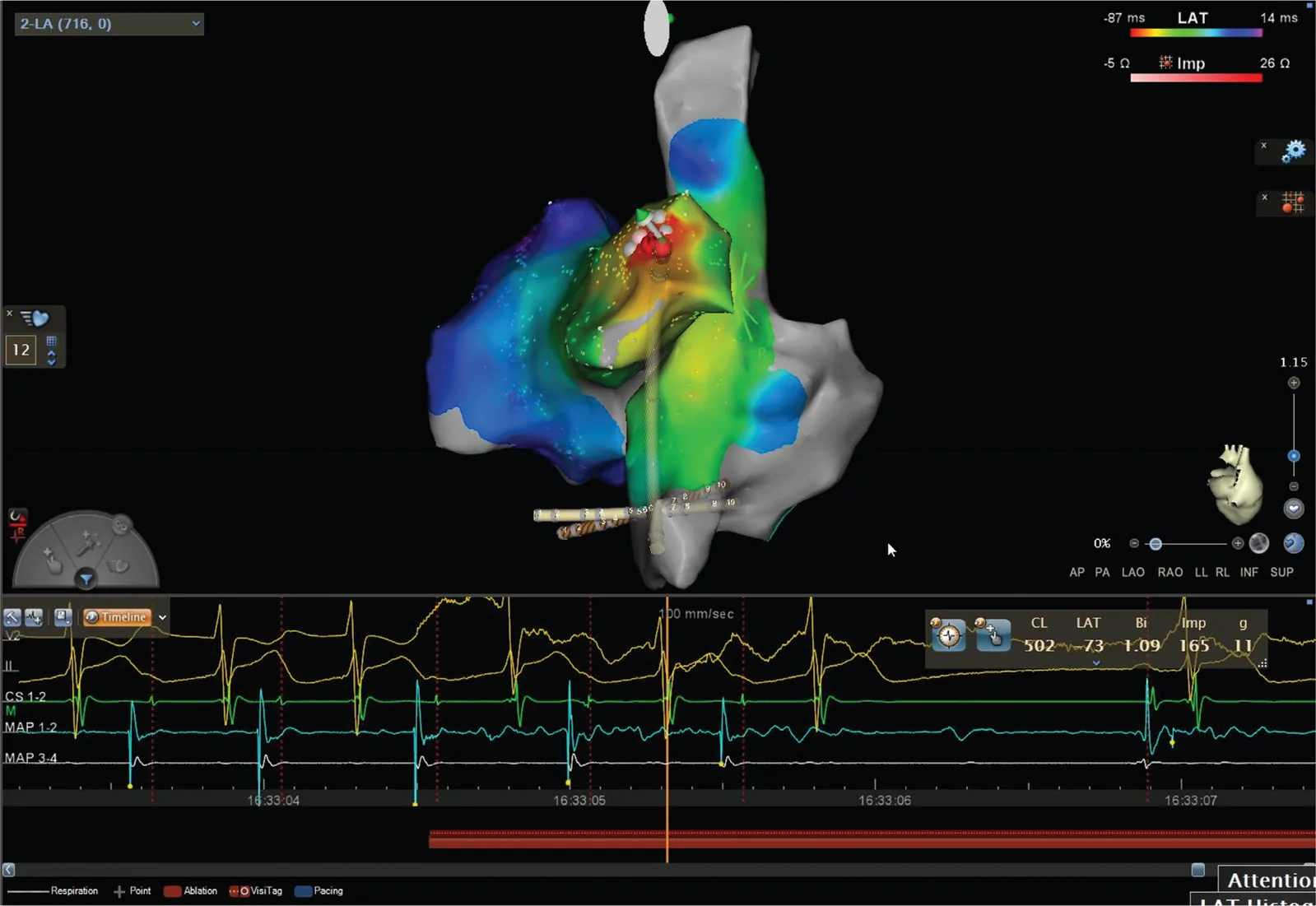
Merged anatomical map showing the location of the successful ablation lesion in the right superior pulmonary vein (red marker) relative to right atrial structures.

Subsequent mapping of the left atrium localized the true focus to the RSPV. As shown in **Figure 3A**, the initial 10 ms of depolarization in the posterior aspect of the RSPV covered a more confined area of 2.1 cm^2^. Ablation here led to successful termination of the tachyarrhythmia in 2 s. The relative anatomical position of the successful ablation site in the RSPV is illustrated in **Figure 4**.

A detailed analysis of the electrograms provides further diagnostic clues. The earliest signal recorded in the RA, while preceding P-wave onset by 28 ms, was a lower-frequency far-field potential followed by a later, sharper near-field component. The corresponding unipolar electrogram displayed a small R-wave **(Figure 2A)**. In contrast, the earliest signal at the successful ablation site within the RSPV was a sharp near-field potential preceding the P-wave by 33 ms, with a QS complex on the unipolar recording **(Figure 3B)**, characteristic of a true focal origin.

### Mechanism of right atrial activation

The observed RA-activation pattern during this RSPV tachycardia is instructive. Activation propagates to the RA primarily via epicardial–myocardial connections between the RSPV and the posteromedial RA. The RA posteromedial epicardial connection is consistently involved in RA activation during focal RSPV tachyarrhythmia, while an additional secondary breakthrough via Bachmann’s bundle (upper interatrial septum) may also be observed in some cases.^2^ The presence of these “intercaval” fibers has been described in postmortem studies.^3^ This differs significantly from sinus rhythm, during which interatrial conduction occurs predominantly via Bachmann’s bundle.

## Conclusion

This case provides the following learning points:

- ***Beware of a large “focal” area.*** During electroanatomic mapping, a diffuse or large area of early activation within a cardiac chamber should raise strong suspicion for a breakthrough site from an adjacent chamber rather than a true focal origin. For RSPV tachycardias manifesting in the RA, a quantitative initial depolarization area of >3.15 cm^2^ is a valuable diagnostic cut-off.
- ***Understand the interatrial connections.*** The most common route of RA activation during focal RSPV tachycardia is via epicardial posteromedial connections, not Bachmann’s bundle. This contrasts with the primary role of Bachmann’s bundle in sinus rhythm.
